# Supplementary material for: Iodine status, household salt iodine content, knowledge and practice assessment among pregnant women in Butajira, South Central Ethiopia
Source: PLoS One. 2022 Nov 28;17(11):e0277208. doi: 10.1371/journal.pone.0277208 (PMC9704620; doi:10.1371/journal.pone.0277208)
Supplement: S1 File — All other data generated in this study was available on the Open Science Frame work (OSF) in the link https://osf.io/c2e79. (DOCX) [file pone.0277208.s001.docx]

# S1 File: Data Collection Format

| **Part I: Socio-demographic characteristics** | | |
| --- | --- | --- |
| 1 | Age |  |
| 2 | Religion | 1.Muslim 2. Orthodox 3. Protestant 4. Others |
| 3 | Ethnicity | 1.Gurage 2. Silte 3.Amhara 4.Oromo 5.Others |
| 4 | Educational qualification | 1. Unable to read andwrite 2. Able to read and write /no formaleducation  3.Grade1-8 4.Grade9-12 5. College andabove |
| 5 | Marital status | 1.Single 2. Married 3.Divorced 4.Widowed |
| 6 | Occupation | 1.Farmer 2.House wife 3.Merchant 4. Student  5.Governmentemployee 6. Others |
| 7 | Total family monthly income |  |
| 8 | How many members are there in your household? |  |
| 9 | Does anyone have a goiter in your family? | 1)yes 2)No |
| **Part II: Clinical examination & Obstetric History** | | |
| 1 | Weight |  |
| 2 | Height |  |
| 3 | hemoglobin/total red blood cell count/ |  |

| 4 | Expected /Gestational time (trimester) | 1)1st 2)2nd 3)3rd |
| --- | --- | --- |
| 5 | How many children do you have? |  |
| **Part III: KAP questions about iodine & iodized salt for pregnant women** | | |
| 1 | Have you ever heard of iodized salt? | 1)Yes 2)No |
| 2 | If your answer is yes for above question, where did you get the information? | 1.Family 2. Neighbors&friends 3. School  4.Healthprofessionals 5. Mass Media (TV, Radio, social medias) 6.Others |
| 3 | Have you ever heard about IDD /Iodine Deficiency Disease /? | 1)Yes 2) No |
| 4 | If your answer is yes for question number 3, what was the source of information? | 1.Family 2. Neighbors&friends 3. School  4.Healthprofessionals 5. Mass Media (TV, Radio social medias) 6. Others.Specify |
| 5 | Do you think everyone should takeiodized salt? | 1)Yes 2)No 3) Don't know/Not sure |
| 6 | If your answer is yes for question number 5, why do you think that we should take iodized salt? |  |
| 7 | What type of salt do you usually buy for daily use? | 1.Iodized 2. Non-iodized 3. Both 4. Don’t know |

| 8 | If the answer for question 7 is iodized salt, how will you identify the iodized salt from the non-iodized? | 1.Cleaner(Whiter) 2. Finer (notcourse)  3.By readingthelabel 4. Other(specify) |
| --- | --- | --- |
| 9 | If the answer is other than 1 for question No 7, why? | 1.Notavailable 2. Moreexpensive  3.Doesn’t knowthebenefit 4. Any other (specify) |
| 10 | How often do you usually buy salt? | 1.Oncea week 2. Every two weeks  3. Onceamonth 4. Any other(specify) |
| 11 | What type of salt are you using right now? | 1.Iodized 2. Non-Iodized  3. don’t know 4. Others(specify) |
| 12 | What the physical state of salt are you using right now? | 1.fine & packed 2. Coarse & non-packed 3. Both 4. Others(specify) |
| 13 | Where do you purchase salt for householduse? | 1.Openmarket 2. Publicdistributionsystem 3. Retailshop  4. Other (specify) |
| 14 | Where do you store the salt in home? | 1.Ontheshelf/table 2. On thefloor 3. Near to fire  4. Getthesunlight 5. Others(specify) |
| 15 | How do you store the salt? (better to observe instead of asking them) | 1.Inplasticbag 2. warped by /In/ paper  3.Samepacket 4. In a container with a lid  5. In a container withouta lid 6. Others (specify) |
| 16 | Did you treat the salt before consuming? | 1.Roasting 2. Washing &drying  3.Dryingby sunlight 4. No treatment atall |
| 17 | when do you usually add salt while cooking? | 1.Atthe beginning 2. In themiddle  3.At theend/onfire/ 4. After cooked & get off thefire |

| 18 | Do you consume Goitrogenic foods such as cabbage? | 1)Yes 2)No |
| --- | --- | --- |
| 19 | If the answer is yes for **no 18**how many times in a week? |  |

# Annex 1: Data Collection Format (Amharic Version)

| **ክፍል1- የስነህዝብናማህበራዊመረጃ** | | |
| --- | --- | --- |
| 1 | እድሜ |  |
| 2 | ሃይማኖት | 1.ኦርቶዶክስ 2. ሙስሊም3. ፕሮቴስታንት4. ሌሎችካሉይጥቀሱ |
| 3 | ብሄር | 1.ጉራጌ 2. ስልጤ3. ኦሮሞ4.አማራ 5. ሌሎችካሉይጥቀሱ |
| 4 | የትምህርትደረጃ | 1.ማንበብና መፃፍአለመቻል2. ማንበብናመፃፍመቻል  3. ከ1ኛ - 8ተኛ የተማረች4.ከ9ኛ - 12ኛ ተማረች5. ኮሌጅ/ዩኒቨርሲቲተማረች |
| 5 | የጋብቻሁኔታ | 1.ያገባች 2. ያላገባች3.የተፋታች 4. ባለቤቷበህይወትየሌለ |
| 6 | ስራ | 1.ገበሬ 2. የቤትእመቤት3. ነጋዴ4. ተማሪ5. የመንግስትሰራተኛ  6. ሌሎችካሉይጥቀሱ------------------------ |
| 7 | የቤተሰብወርጠቅላላገቢ |  |
| 8 | የቤተሰብአባላትብዛት |  |
| 9 | በቤትውስጥእንቅርትያለበትሰውአለ? | 1. አለ 2.የለም |
| **ክፍል2 - የክሊኒክምርመራናየእርግዝናሁኔታመረጃ** | | |
| 1 | ክብደት |  |
| 2 | ቁመት |  |

| 3 | የሄሞግሎቢንመጠን(ጠቅላላየቀይደምሴልቁጥር) |  |
| --- | --- | --- |
| 4 | አሁንየሚገኙበትየእርግዝናወር(ወቅት) | 1.የመጀመሪያትራይሚንስተር 2.ሁለተኛትራይሚንስተር  3.ሶስተኛትራይሚንስተር |
| 5 | ስንትልጆችአሉሽ? |  |
| **ክፍል3 - አዮዲንንናየጨውአጠቃቀምናየተመለከቱጥያቄዎች** | | |
| 1 | አዮዲንስላለበትጨውሰምተውያውቃሉ | 1.አውቃለሁ 2. አላውቅም(ከላይለሚገኘውጥያቄመልሶ2ቁጥርከሆነወደ7ተኛው ጥያቄይለፉ) |
| 2 | ለመጀመሪያውጥያቄመልሶ1 ቁጥርከሆነመረጃውንከየትአገኙት? | 1.ከቤተሰብ 2. ከጓደኛወይምከጎረቤት3. ከትምህርትቤት4. ከጤናባለሙያዎች5. ከመገናኛብዙሃን(ቲቪ፤ሬድዮ፤ማህበራዊድረ-ገፅ) 6. ሌሎችካሉይጥቀሱ |
| 3 | በአዮዲንእጥረትምክንያትስለሚመጡበሽታዎችሰምተውያውቃሉ | 1. አውቃለሁ 2.አላውቅም |
| 4 | ለሶስተኛውጥያቄመልሶ1 ቁጥርከሆነከየትነውየሰሙት? | 1. ከቤተሰብ2. ከጓደኛወይምከጎረቤት3. ከትምህርትቤት4.ከጤናባለሙያዎች5. ከመገናኛብዙሃን(ቲቪ፤ሬድዮ፤ማህበራዊድረ-ገፅ) 6. ሌሎችካሉይጥቀሱ |
| 5 | ሁሉም ሰው አዮዲን ያለበት ጨውመውሰድአለበትብለውያስባሉ? | 1. አዎ 2.የለበትም 3. አላውቅም |

| 6 | ለ5ተኛው ጥያቄመልሶአዎከሆነለምንአዮዲንያለውጨውመመገብአለብንብለውያስባሉ? |  |
| --- | --- | --- |
| 7 | ለምግብነትየሚገዙትጨውምንአይነትነው? | 1.አዮዲንያለበት 2.አዮዲንየሌለበት 3. ሁሉቱንም/ያገኘሁትን/  4. አላውቅም |
| 8 | ለ7ተኛው ጥያቄመልሶ1 ቁጥርከሆነአዮዲንያለበትንጨውከሌለበትእንዴትይለዩታል? | 1. የበለጠነጭ(ንፁህ)ነው 2.ደቃቅነው 3. ማሸጊያውላይየሚገኘውንፅሁፍበማንበብ 4. ሌላካለይጥቀሱ |
| 9 | ለ7ተኛው ጥያቄመልሶ ከ 1 ቁጥርውጪከሆነለምንአዮዲንያለበትንጨውአይገዙም? | 1. በቅርብስለማገኝ2. ውድስለሆነ3. ጥቅሙንስለማላውቅ4. ሌላካለይጥቀሱ |
| 10 | በምንያህልጊዜጨውይገዛሉ? | 1. በየሳምንቱ2. በየሁለትሳምንቱ3. በየወሩ4. በየ6 ወር5. ሌላካለይጥቀሱ |
| 11 | በአሁኑሰአትየትኛውንየጨውአይነትእየተጠቀሙይገኛሉ? | 1. አዮዲንያለበት2. አዮዲንየሌለበት3. ያገኘሁትን(ማንኛውምአይነት) 4. ሌላካለይጥቀሱ |
| 12 | በአሁኑሰአትየትኛውንየጨውአይነትእየተጠቀሙይገኛሉ? | 1.ደቃቅናየታሸገ2.ጠጣርናብትን 3.ያገኘሁትን 4. ሌላካለይጥቀሱ |
| 13 | ለምግብነት የሚጠቀሙበትን ጨውየሚገዙትከየትነው? | 1. ከጉልትገበያ2. ከሸማቾችማህበር3. ከሱቅ4. ሌላካለይጥቀሱ |

| 14 | ለምግብነትየሚጠቀሙበትንጨውየሚያስቀምጡትየትነው? (በምልከታየሚሞላ) | 1. መደርደሪያወይምጠረ’ዛላይ2. መሬትላይ 3. ከእሳትአጠገብ  4. ፀሀይየሚያገኘውቦታ4. ሌላካለይጥቀሱ |
| --- | --- | --- |
| 15 | ለምግብነትየሚጠቀሙበትንጨውበምንውስጥነውየሚያስቀምጡት? ( በምልከታየሚሞላ) | 1. በማዳበሪያወይምበላስቲክ2. በወረቀት3. እንደታሸገ(በማሸጊያውውስጥ) 4. ክዳንባለውእቃ5. ክዳንበሌለውእቃ6. ሌላካለይጥቀሱ |
| 16 | ለምግብነትየሚጠቀሙበትንጨውጥቅምላይከማዋሎበፊትምንያደርጉታል? | 1. በእሳትመቁላት2. ማጠብናማድረቅ3. ፀሀይላይማስጣት4.  ምንምአናደርገውም |
| 17 | ምግብ በሚያበስሉበት ወቅት ጨውየሚጨምሩትመቼነው? | 1. መጀመሪያላይ2. መሀልላይ3. ከእሳትሊወጣሲል4. ከእሳትከወረደበኃላ |
| 18 | ለእንቅርትተጋላጭየሚያደርጉምግቦችንማለትምጥቅልጎመንወይምካሳቫይመገባሉ? | 1. አዎ 2.አልመገብም |
| 19 | ለ18ኛው ጥያቄመልሶአዎከሆነበሳምንትስንትጊዜይመገባሉ? |  |

**Annex 2: Consent Form**

My name is Senait Tadesse. Currently, I am working my master’s degree in Pharmaceutical Analysis &Quality Assurance in Addis Ababa University. As part of my Master’s program, I am doing my thesis entitled as “iodine status of pregnant women in Butajira, Ethiopia; BUNMAP cohort”.Wewanttostudyinwhatextentpregnantwomenarevulnerabletoiodine deficiencyinthisarea.Wealsowanttostudywhetherthesalttheyareusingissufficientlyiodized or not & thus to identify whether the iodized salt is sufficient iodine source for them or additional supplement is necessary. Deficiency of iodine during pregnancy could cause adverse health effect both on the fetus & mother. To know this, we need your help and would like to ask you some questions. We would alsotestthesaltconsumedbyyouandanalyzeyoururinetoseeifthereisanydeficiencyofiodine. We will not perform any other test on your urine. The study result will be used as information source & to recommend the stake holders for future planning andintervention.Weassureyouthat all the information that we get from you including the reports of test results will be confidential and will not be disclosed to anyone except you. your name will not be written on this form. We will only use a code number instead. Your name will not appear in any report of the surveytoo.

We would also like to inform you that if you agree to take part in this survey your participation will be voluntary. No incentive will be given to you for taking part in this study. You can choose not to take part. You can choose not to answer a specific question. You can also withdraw from this study anytime you wish to stop. You are also at liberty to refuse to give your salt or urine sampleifyoudon'tlike.Thiswillnotaffectyourrightstohealthcareservice.Thereisnospecific risktoyouexceptforspendingyourvaluabletimeforansweringthequestions.

If you wish to find out more about this survey before taking part, you can ask any questions you want. You can also contact me at phone no. 0913346183 or my advisor Mr. AyenewAshnef (Assistant professor) at 0920558470. If you are agreeing to take part, we will go ahead now and sign this paper. By signing, you will declare and consent that “I have read the foregoing information,orithasbeenreadtome.Ihavehadtheopportunitytoaskquestionsaboutitandany questionsIhaveaskedhavebeenansweredtomysatisfaction”.Iconsentvoluntarilytoparticipate in this study and understand that I have the right to withdraw from the study at any time without in any way affecting my health care in the future.

Name of the interviewerSignature/thumb of theparticipant

# Annex 2: Consent Form (Amharic Version)

ሰናይትታደሰእባላለሁ፡፡በአዲስአበባዩኒቨርሲቲበፋርማሲየሁለተኛድግሪዬንእየሰራሁእገኛለሁ፡፡የምርምርስራዬምበእርጉዝሴቶችየአዮዲንመጠንእናየጨውአጠቃቀምላይነው፡፡ ነፍሰጡርሴቶችበእርግዝናወቅትበቂየአዮዲንንጥረነገርካላገኙለተለያዩየጤናችግሮችይጋልጣሉ፡፡በተጨማሪምፅንሱለብዙየጤናእክሎችይጋለጣል፡፡ስለዚህምበዚህአካባቢየሚኖሩነፍሰጡርሴቶችለአዮዲንእጥረትያላቸውንተጋላጭነትለማጥናትእንሞክራለን፡፡በተጨማሪምለምግብነትየሚጠቀሙትንጨውበቂየአዮዲንመጠንመያዙንእንመረምራለን፡፡ይህንንምለማጥናትየእናንተቀናትብብርያስፈልገናል፡፡ስለሆነምለምንጠይቃቹጥያቄዎችተገቢውንምላሽእንድትሰጡንበትህትናእንጠይቃለን፡፡ በተጨማሪምየሽንትናየጨውናሙናእንወስዳለን፡፡በናሙናዎቹውስጥያለውንየአዮዲንመጠንብቻእንለካለን፡፡የጥናትውጤቱምወደፊትለሚደረጉየጤናፕሮግራሞችናጥናቶችእንደግብአትያገለግላል፡፡በዚህጥናትመሳተፍበሙሉፍቃደኝነትላይየተመሰረተሲሆንምንምአይነትክፍያወይምጥቅማጥቅምአይኖረውም፡፡በጥናቱአለመሳተፍምሆነየተወሰኑጥያቄዎችንአለመመለስወይምበፈለጉትጊዜጥናቱንአቋርጦመውጣትይቻላል፡፡ይህምየሚያገኙትንየጤናአገልግሎትበምንምአይነትሁኔታአያስተጓጉልም፡፡ማንኛውምከርሶየሚገኝመረጃበሙሉሚስጥራዊነቱእንደሚጠበቅእናረጋግጥሎታለን፡፡የእርሶስምየትኛውምአይነትየጥናትውጤትላይአይገለፅም፡፡ሁሉምመረጃየሚመዘገበውበሚስጥርቁጥርብቻይሆናል፡፡በተጨማሪስለጥናቱማወቅከፍለጉበሚከተሉትስልክቁጥሮችመደወልይችላሉ፡፡(0913346183-ሰናይት ታደሰ ፤0920558470-ረዳት ፕሮፌሰርአየነውአሸነፍ፡፡)በጥናቱላይለመሳተፍከተስማሙጥናቱንመቀጠልእንችላለን፡፡

የምፈልገውንጥያቄመጠየቅናመልሱንምየማግኘትእድልተሰጥቶኛል፡፡ከላይያሉትንመረጃዎችበሙሉአንብቤቸዋለሁወይምተነቦልኛል፡፡በፈለኩትጊዜጥናቱንአቋርጬመውጣትእንደምችልናየማገኘውየጤናአገልግሎትላይምንምአይነትችግርእንደማገጥመኝተረድቻለሁ፡፡ስለዚህምበራሴፍቃድበጥናቱላይለመሳተፍተስማምቻለሁ፡፡

የተሳታፊዋፊርማ ---------------- የጠያቂውስም---------------------
